# An Affinity–Effect Relationship for Microbial Communities in Plant–Soil Feedback Loops

**DOI:** 10.1007/s00248-013-0349-2

**Published:** 2014-01-09

**Authors:** Yi Lou, Sharon A. Clay, Adam S. Davis, Anita Dille, Joel Felix, Analiza H. M. Ramirez, Christy L. Sprague, Anthony C. Yannarell

**Affiliations:** 1 Department of Natural Resources and Environmental Sciences, University of Illinois at Urbana–Champaign, 1102 S. Goodwin Avenue, Urbana, IL 61801 USA; 2 Department of Plant Science, South Dakota State University, Brookings, SD USA; 3 USDA-ARS, Global Change and Photosynthesis Research Unit, Urbana, IL USA; 4 Department of Agronomy, Kansas State University, Manhattan, KS USA; 5 Department of Crop and Soil Science, Oregon State University, Corvalis, OR USA; 6 Department of Plant, Soil and Microbial Sciences, Michigan State University, East Lansing, MI USA

## Abstract

Feedback loops involving soil microorganisms can regulate plant populations. Here, we hypothesize that microorganisms are most likely to play a role in plant–soil feedback loops when they possess an affinity for a particular plant and the capacity to consistently affect the growth of that plant for good or ill. We characterized microbial communities using whole-community DNA fingerprinting from multiple "home-and-away" experiments involving giant ragweed (*Ambrosia trifida* L.) and common sunflower (*Helianthus annuus* L.), and we looked for affinity–effect relationships in these microbial communities. Using canonical ordination and partial least squares regression, we developed indices expressing each microorganism's affinity for ragweed or sunflower and its putative effect on plant biomass, and we used linear regression to analyze the relationship between microbial affinity and effect. Significant linear affinity–effect relationships were found in 75 % of cases. Affinity–effect relationships were stronger for ragweed than for sunflower, and ragweed affinity–effect relationships showed consistent potential for negative feedback loops. The ragweed feedback relationships indicated the potential involvement of multiple microbial taxa, resulting in strong, consistent affinity–effect relationships in spite of large-scale microbial variability between trials. In contrast, sunflower plant–soil feedback may involve just a few key players, making it more sensitive to underlying microbial variation. We propose that affinity–effect relationship can be used to determine key microbial players in plant–soil feedback against a low "signal-to-noise" background of complex microbial datasets.

**Electronic supplementary material:**

The online version of this article (doi:10.1007/s00248-013-0349-2) contains supplementary material, which is available to authorized users.

## Introduction

Plant–soil feedback (PSF) is a term used to describe an interactive loop involving plants and the biological, chemical, and physical properties of the soil [1]. In its most basic form, PSF involves a plant whose growth alters some aspect of the soil system, and this alteration has consequences for future plant performance in that patch of soil [2]. For example, a plant may attract root pathogens and parasites, raising the local population density of these organisms, and this "negative feedback" will serve to suppress the future performance of the plant or its offspring [3]. Soil microorganisms are regarded as important agents of PSF, and there has been great interest in exploring microbial capacity to generate PSF in a number of different systems [4–10].

Bever [11] introduced the framework of microbial PSF in a two-plant system that included the effects of plant competition. He reasoned that each plant must respond to its own feedback microorganisms and also those of its neighbor(s). Modeling these interactions shows that certain kinds of PSF can allow for the coexistence of competing plant species, counteracting the tendency of competitive exclusion [11]. As is necessary for modeling, Bever's model greatly simplifies the way that the microbial community is portrayed. Rather than consisting of a specified, dynamic set of taxa, Bever's microbial community is represented in the abstract as a fluctuating point moving along an axis that separates each plant's singular influence on the microbial community to opposite ends [11]. This mathematical abstraction was integral to the foundational theoretical work on PSF, but it also makes it difficult to study the specific interactions that occur between plants and individual species of the belowground community.

Because PSF is driven by these species-to-species interactions, with all of their attendant phenotypic and phylogenetic variability, the identity of the particular microbial groups involved should matter for PSF. To date, much work on PSF has focused on a handful of microbial groups that are known to interact strongly with plants: mycorrhizal fungi [6, 8, 12–14], nitrogen-fixing rhizobacteria [15–18], and plant pathogens [3, 6, 19, 20]. However, there are additional direct and indirect channels by which soil microorganisms may impact plant performance. For example, microorganisms may control decomposition and thereby influence nutrient availability [21, 22]; they may produce compounds that affect plant growth and mortality [23–26]; they may be involved in "nutrient mining" at the root tips of plants [27, 28]; and they may interact with each other to support or suppress the growth of other soil microorganisms that benefit or harm plants directly [29, 30]. Given the vast taxonomic diversity of soil microorganisms [31], it is likely that a number of important microbial agents of PSF are yet to be discovered, and narrow approaches based on specific microbial taxa may miss these important but little-known interactions.

Modern DNA-based approaches to microbial community ecology can survey whole microbial communities in unprecedented detail and thus may facilitate the discovery of important new plant–microbe interactions. However, the application of whole-community approaches to study microbial communities involved in PSF have thus far provided mixed results. Bacterial community composition often shows no correlation — or a very weak correlation — with the plant species treatments used to set up PSF experiments [32–34], while fungal community composition sometimes shows a response [8, 33–35]. These results highlight the shortcomings of whole-community approaches: they may be too broad in their focus. Thus, while it is almost certainly useful to look beyond the small set of functional groups that have thus far received the most attention, it is not reasonable to assume that every microorganism in the soil community is participating in a PSF loop in a meaningful way. Indeed, if a few key species are involved in the PSF, the remainder of the microbial community will contribute unrelated variance, leading to weak/non-significant multivariate responses. Whole-community approaches open up a new space for discovery, but they also present a "signal-to-noise" problem when presented with microbial communities where a majority of taxa do not participate directly in PSF.

The nature of feedback loops provides a potential solution to this signal-to-noise problem. For a system to constitute a *feedback loop*, the various components of the system must respond to each other and also alter each other. For example, while soil pH can alter plant community composition through differential fitness impacts on different plant species, a plant–pH feedback loop will only exist when the plants also alter soil pH. Similarly, for a soil microorganism to be engaged in a feedback loop with plants, it must both provide differential fitness effects on different plant species, and it must differentially respond to the presence/abundance of different plant species. Microorganisms that do not asymmetrically associate with one plant or the other (i.e., express affinity) will not be able to respond to changes in plant density, and therefore are not joined in a feedback loop to the plants. Similarly, microorganisms that do not consistently harm or benefit the plant (i.e., produce a characteristic effect) will not provide coherent feedback as the above- and belowground communities change. Therefore, we propose that microbial taxa possessing both of these characteristics have the greatest potential for regulation of a PSF system.

We propose that the search for specific microorganisms with the potential to participate in feedback loops (hereafter, "PSF microorganisms") can be accomplished in a whole-community context through a quantitative consideration of two traits that PSF microorganisms in a two-plant interaction must possess: they must have both (1) an affinity for one plant over the other and (2) an effect (either positive or negative) on the growth or fitness of at least one of the plants. Furthermore, we argue that microbial regulation of the PSF system will be most effective when there is a relationship between affinity and effect across the entire microbial community, such that the microbes with the strongest affinity for a plant also produce the strongest (positive or negative) effect on plant growth, and the positive and negative effects of high-affinity microbes do not tend to cancel each other out.

Here we use a series of standardized "home-and-away" trials carried out in multiple locations across the north central United States to characterize affinity–effect relationships for soil microorganisms associated with two early successional plants. We hypothesize that microorganisms participating in PSF loops will possess both an affinity for a particular plant and the capacity to strongly affect the growth of that plant, giving rise to positive or negative PSF dependent on the direction of the effect.

## Materials and Methods

### Home-and-Away Trials

Ten independent "home-and-away" [36] trials were conducted using two agricultural weeds, common sunflower (*Helianthus annuus* L.) and giant ragweed (*Ambrosia trifida* L.). All trials used common seed stocks gathered from Manhattan, Kansas (sunflower) and Urbana, Illinois (ragweed). Trials were conducted independently at agricultural research facilities in Michigan, Illinois, Kansas, South Dakota, and Oregon using local soils gathered on site. Two independent trials with staggered start times were run at both Illinois and Kansas; in Michigan, soils were collected from two different farm fields (near St. Charles and East Lansing), and each of these soils were used in two separate trials with staggered start times, for a total of four Michigan trials. All other sites conducted a single trial each. We treat each of these trials as (*n* = 10) independent experimental runs for the purpose of this study.

Specific details about soil properties and management histories of sites have been previously described [36], along with an analysis of plant biomass response to the "home-and-away" treatments. Briefly, for each trial, local soil was gathered, mixed 50:50 with sand, and distributed into 40 pots (30 cm [diameter] × 30 cm [height]) in a greenhouse. Sunflower seeds were introduced to 20 of these pots, and ragweed seeds were introduced to the other 20. Plants were allowed to germinate and grow for 10 weeks, after which time the entire plant was harvested. This procedure was repeated, with new sunflower or ragweed seeds being introduced to their respective pots, followed by 10 weeks of growth and plant harvesting. Following these two 10-week periods of conditioning by either sunflower or ragweed, ten pots with sunflower-conditioned soil were replanted with sunflower seeds (the "home" treatment), and ten pots with sunflower-conditioned soil were planted with ragweed seeds (the "away" treatment). Ragweed-conditioned soils were similarly planted with sunflower or ragweed seeds to comprise "away" and "home" treatments. These plants were allowed to grow for 10 weeks in their "home" or "away" soils, and then plants were harvested for dry biomass determination (48 h of drying at 60°C). After harvesting plant shoots and roots, the remaining soil from each pot was collected into separate Ziploc bags and stored at −20°C for microbial community analysis.

The results of these trials showed that ragweed experienced consistent negative PSF, while sunflower PSF was negative or positive, depending on the trial [36]. A bioassay for potential allelopathic effects [37], conducted using *Lactuca sativa* seeds exposed to conditioned soil under optimum germination conditions for 7 days, did not support allelopathic suppression as a mechanism behind this observed PSF. Therefore, we investigated potential microbial involvement as described below.

### Microbial Community Composition

Ziploc bags containing soil from each pot were thawed and homogenized by shaking. We used a sterile (70 % EtOH) metal scoop to collect a 0.5-g subsample of soil from each bag, and Bulk DNA was extracted from these subsamples using the FastDNA Spin kit for Soil (MP Biomedicals, Solon, OH) following the manufacturer's instructions. Additional purification of DNA was accomplished with a 15-min incubation at 65°C with 1 % cetyltrimethylammonium bromide and 0.7 M NaCl, followed by a 24:1 chloroform/isoamyl alcohol extraction to remove impurities bound to the cetyltrimethylammonium bromide in the organic fraction.

Microbial community composition was characterized by automated ribosomal intergenic spacer analysis (ARISA), a length heterogeneity polymerase chain reaction (PCR)-based approach to rapidly generate whole-community "fingerprints" of bacterial and fungal assemblages [38, 39]. ARISA uses universal primers to amplify the ITS region of bacterial ribosomal RNA operons [38] or the equivalent ITS1-5.8S-ITS2 region in fungi [39]. Because these hypervariable stretches of "junk DNA" vary in length across taxonomic groups (roughly the strain, species, or genus level), the collection of ARISA amplicons produced from a soil sample represents the taxonomic diversity and composition of that sample. Bacterial ARISA used primers 1406 F and 23SR [38], and fungal ARISA used primers 2234C and 3216 T [39]; the 5′ ends of primers 1406 F and 3216 T were labeled with fluorochrome dyes (6-FAM and HEX, respectively), to permit detection of ARISA amplicons during capillary gel electrophoresis. Each 50-μl PCR reaction contained 2 ng of template DNA (from soil samples), 5 mM Tris–HCl (pH 8.3), 0.25 mg/ml bovine serum albumin, 2.5 mM MgCl_2_, 0.25 mM of each deoxynucleoside triphosphate, 0.4 μM of each primer, and 1.25 U of Taq polymerase (Promega, Madison, WI, USA). PCR cycling conditions included an initial denaturation at 94°C for 2 min, followed by 26 cycles of 94°C for 35 s, 55°C for 45 s, and 72°C for 2 min, with a final 72°C extension for 2 min. Separation and detection of ARISA amplicons was performed under denaturing conditions on an ABI 3730XL Genetic Analyzer (Applied Biosystems, Carlsbad, CA, USA) with an internal ROX1000 size standard (BioVentures, Inc., Murfreesboro, TN, USA).

Taxonomic characterization of microbial communities was based on ARISA amplicon length (base pairs). Within the range of 400–1,000 bp for bacteria [38] or 300–1,000 bp for fungi [39], all ARISA amplicons of the same size, after accounting for statistical variation in size calling, were taken to represent a single microbial taxon. Because the taxonomic resolution of ARISA does not correspond to any single level of the Linnaean classification scheme, we refrain from talking about microbial "species", and we hereafter follow the standard microbial ecology practice of referring to these ARISA-defined groups as operational taxonomic units (OTUs). Size calling and definition of same-size "bins" for bacterial and fungal OTUs used GeneMarker v. 1.95 (SoftGenetics, LLC, State College, PA, USA), with manual correction of the software-defined OTU bins in order to eliminate any overlapping bins. Every ARISA amplicon in each sample was assigned to an OTU bin, and the signal intensity (peak height) of each amplicon was taken to reflect the abundance of that OTU in the sample. Across all samples, the collection of OTUs and their signal intensities constitute a sample-by-OTU data table analogous to the sample-by-species tables that are typical of community ecology studies [40].

### Analyzing the Affinity–Effect Relationship

Multivariate data analyses were used to assign three indices to every ARISA OTU. The first index was intended to represent the affinity of each microbial OTU for either ragweed or sunflower. The second and third indices were used to represent the effects of each OTU on ragweed and sunflower, respectively. These indices were created based on OTU-specific patterns of (relative) abundance and distribution across different combinations of pots from the "home-and-away" trials. Bacterial and fungal indices were assigned separately, and index assignment was conducted separately for each trial (in Oregon, South Dakota, etc.). That is, ten sets of indices were assigned to the bacterial OTUs encountered in the different trials, and ten separate sets of indices were assigned to the fungal OTUs. For all data analyses, the raw ARISA OTU data were subject to the Hellinger transformation [41] to eliminate run-to-run variability from the Genetic Analyzer; thus, the intensity of ARISA peaks represents "relative abundance" of each OTU in a single pot soil sample.

The affinity index was created using data from the ragweed "home" and sunflower "home" pots, because soil microbial communities in these pots had undergone three successive rounds of selection by these plant species. The Hellinger-transformed ARISA data were used to create pot-by-pot Bray–Curtis dissimilarity matrices for each trial, and these matrices were used for community ordinations by Canonical Analysis of Principal Coordinates (CAP) [42], using the plant species identity as the constraining variable. Thus, the first (and only) canonical axis placed ragweed-associated communities on one side of the axis and sunflower-associated communities on the other. For consistency of data presentation, we placed ragweed on the left (negative) side of the first CAP axis and sunflower on the right (positive) side, and we therefore multiplied some of the eigenvectors by −1 in order to achieve this arrangement. We then fit the OTUs onto this axis using weighted averages of their community CAP axis score and their relative abundance in the sample-by-OTU table [43]. We used these fitted OTU scores on the first CAP axis as the index of affinity for each OTU. Thus, OTUs with negative values for this index were highly associated with ragweed, and those with positive scores were highly associated with sunflower. CAP and weighted averaging were performed in the R statistical environment using the "capscale" function of package "vegan" [43].

The ragweed and sunflower effect indices were created using data from "home" and "away" pots for ragweed or the "home" and "away" pots for sunflower. To create these axes, we modeled plant biomass as a function of microbial community composition using Projection to Latent Structure Regression (PLSR; also commonly known as partial least squares regression [44]). Conceptually, PLSR is similar to using a Principal Components Analysis ordination of (multivariate) community data in order to construct a set of orthogonal "latent variables" representing variation in community composition; these latent variables are then used as independent variables in regressions against a response variable. However, rather than constructing latent variables that explain the maximum variance in the community data (as in Principal Coordinates Analysis), the ordination in PLSR creates latent variables that maximize the covariance between the community data and the response variable [44]. We used the Hellinger-transformed ARISA data to create the latent variables, and the total dry biomass of the ragweed (or sunflower) plants in the pots were used as response variables. Thus, the first latent variable in the PLSR expressed microbial community turnover that was most associated with variation in plant biomass. We used the loadings of OTUs along the first PLSR latent variable as the index of effect. Thus, OTUs with high values for this index for ragweed tended to be found in pots with large ragweed biomass, and those with low values were found associated with smaller ragweed plants. PLSR was performed in R using function plsr() in the package "pls" [45].

Every bacterial and fungal OTU in each trial received an index of affinity, an index of effect on ragweed, and an index of effect on sunflower. We conducted separate linear regressions of effect versus affinity for each trial using the R function glm(). In addition, we identified potentially important OTUs for PSF using the distribution of affinity and effect scores within each trial. We considered an OTU to be potentially important for PSF if it was in the upper or lower 2.5 % tails of both the affinity and effect indices for a given trial. Thus, we determined which OTUs diplayed both the strongest affinity and the largest beneficial or harmful effect. We summed the number of potentially important OTUs for ragweed and sunflower in each trial, and then we used a paired *t*-test to determine if similar numbers of OTUs were associated with each plant.

### Generalized Microbial Community Responses

To explore overall patterns in microbial community composition, we used Nonmetric Multidimensional Scaling. We also tested for significant shifts in microbial community composition across different trials and in response to "home" and "away" treatments using Permutational Multivariate Analysis of Variance (perMANOVA) [46]. For each of these analyses, we considered each pot (*n* = 40 pots per trial) to be an independent experimental unit, and thus we were able to statistically assess the impact of trial (ten levels), plant species in the initial "training" phase, plant species in the final phase, and all two- and three-way interactions using a three-way perMANOVA design with 400 total observations. These analyses both used the Bray–Curtis dissimilarity matrix of Hellinger-transformed ARISA data, with statistical significance assessed through 1,000 permutations of the ARISA data, using functions metaMDS() and adonis() from package "vegan" in R [43].

## Results

**Fig. 1:**
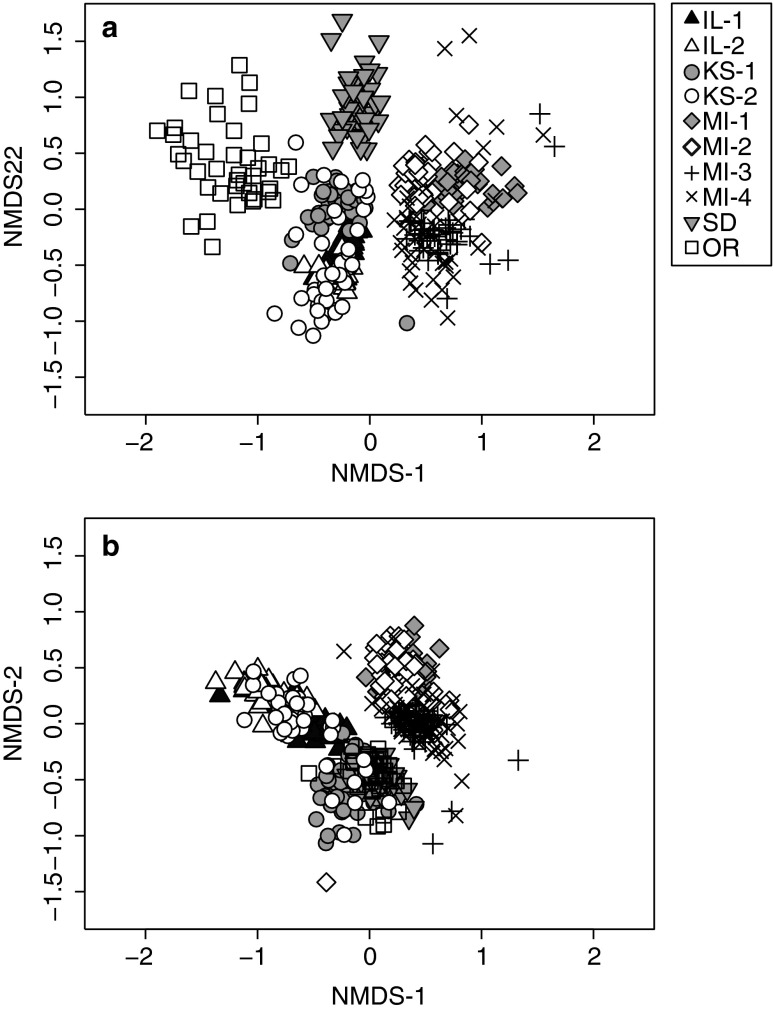
Nonmetric multidimensional scaling plots for microbial communities in "home-and-away" trials. Bacterial (**a**) and fungal (**b**) community composition was assessed from pots in the final stage of the ten "home-and-away" trials (i.e., after exposure to three different generations of plants). Final two-dimensional stress is 0.21 and 0.20 for panels **a** and **b**, respectively

The composition of bacterial and fungal communities was broadly different across the different states performing the "home-and-away" trials (Fig. 1). In states performing multiple trials (Illinois, Kansas, Michigan), community composition also tended to vary from trial to trial, particularly for soil fungi (Fig. 1b). In support of these observations, "trial" explained the largest component of bacterial and fungal community composition according to perMANOVA (Table 1). Across the entire dataset, plant identity in the soil training phase and in the final experimental phase were found to have statistically significant but very weak influences on microbial community composition (Table 1). Thus, plants were exposed to different communities of microorganisms in the different "home-and-away" trials, and they selected for different microorganisms from these different starting species pools.

**Table 1:** Permutational MANOVA shows the treatment effects on soil microbial community composition in the "home-and-away" trials

	*df*	*F* ^a^	*R* ^2^	*p* ^b^
*Response: bacterial communities*				
Trial^c^	9	28.88	0.373	0.001***
Training plant^d^	1	3.03	0.005	0.001***
Final plant^e^	1	4.04	0.006	0.001***
Trial:Training plant	9	3.05	0.039	0.001***
Trial:Final plant	9	2.84	0.037	0.001***
Training plant:Final plant	1	2.43	0.003	0.003**
Trial:Training plant:Final plant	9	2.25	0.030	0.001***
*Response: fungal communities*				
Trial^c^	9	35.5	0.412	0.001***
Training plant^d^	1	5.74	0.007	0.001***
Final plant^e^	1	4.04	0.005	0.001***
Trial:Training plant	9	4.21	0.049	0.001***
Trial:Final plant	9	2.93	0.034	0.001***
Training plant:Final plant	1	2.70	0.003	0.005**
Trial:Training plant:Final plant	9	2.55	0.030	0.001***

^a^This is actually a "pseudo F" based on decomposition of the response and residual matrices (McArdle and Anderson 2001)

^b^Significance codes: ***alpha <0.001: **alpha <0.01

^c^Accounts for variation between the ten "home-and-away" trials

^d^Accounts for variation due to the plant species (ragweed or sunflower) in the initial soil training stages of the "home-and-away" trials

^e^Accounts for variation due to the plant species (ragweed or sunflower) in the final stage of the "home-and-away" trials

The dynamics of a minority of the soil microbial taxa were often sufficient to capture a significant portion of variation in plant performance (Table 2). Indices of affinity from the individual "home-and-away" trials explained from 9 % to 37 % of variation in microbial community composition (Table 2), and although there was much variation in the strength of microbial affinity for ragweed or sunflower across the trials, most of the indices of affinity accounted for less than 20 % of total microbial community variability. In line with this result, indices of effect were driven by a small proportion of microbial community variability (Table 2); these numbers ranged from 5 % to 25 %, but most of them captured less than 10 % of the total microbial variability in a trial. However, the indices of effect were strongly related to variation in plant biomass (Table 2), with most of them capturing over 70 % of the variation in plant performance.

**Table 2:** Variance in microbial community composition and plant biomass attributable to indices of affinity^a^ and effect^b^

Trial	Microbial group	Affinity variance (%)^a^	Plant	Effect variance (%)^b^	
				Microbial community^c^	Plant biomass^d^
IL-1	Bacteria	13.3	Ragweed	8.6	58.6
			Sunflower	6.8	85.5
	Fungi	9.2	Ragweed	10.5	66.4
			Sunflower	12.7	62.6
IL-2	Bacteria	12.6	Ragweed	12.6	60.7
			Sunflower	9.5	58.5
	Fungi	9.3	Ragweed	16.4	84.7
			Sunflower	10.5	59.0
KS-1	Bacteria	18.7	Ragweed	8.1	77.9
			Sunflower	8.4	78.0
	Fungi	22.5	Ragweed	12.1	64.7
			Sunflower	10.0	74.4
KS-2	Bacteria	22.8	Ragweed	19.0	51.0
			Sunflower	6.4	84
	Fungi	20.9	Ragweed	20.7	44.3
			Sunflower	23.5	41.3
MI-1	Bacteria	15.2	Ragweed	16.5	58.3
			Sunflower	7.7	80.6
	Fungi	18.8	Ragweed	13.4	63.0
			Sunflower	8.2	87.2
MI-2	Bacteria	13.4	Ragweed	10.7	76.3
			Sunflower	10.0	72.6
	Fungi	20.7	Ragweed	15.2	68.5
			Sunflower	7.3	81.4
MI-3	Bacteria	10.4	Ragweed	9.7	80.2
			Sunflower	6.1	83.7
	Fungi	14.8	Ragweed	9.0	79.4
			Sunflower	5.7	79.3
MI-4	Bacteria	15.0	Ragweed	8.0	81.0
			Sunflower	9.6	73.6
	Fungi	12.7	Ragweed	12.9	70.5
			Sunflower	17	48.8
OR	Bacteria	18.7	Ragweed	11.4	84.2
			Sunflower	12.8	69.3
	Fungi	37.3	Ragweed	10.7	68.8
			Sunflower	8.0	77.9
SD	Bacteria	9.4	Ragweed	8.8	67.9
			Sunflower	5.8	90.7
	Fungi	22.6	Ragweed	10.0	65.0
			Sunflower	9.1	84.6

^a^Affinity index and percent variance is derived from canonical analysis of principal coordinates. The affinity variance percentage expresses the turnover of microbial communities due to affinity for either ragweed or sunflower

^b^Effect index and percent variance derived from the first axis of partial least squares regression

^c^Expresses the covariation of microbial communities with plant biomass, as a percentage of total microbial variance

^d^Expresses the plant biomass variance explained by covariation with microbial community composition

**Fig. 2:**
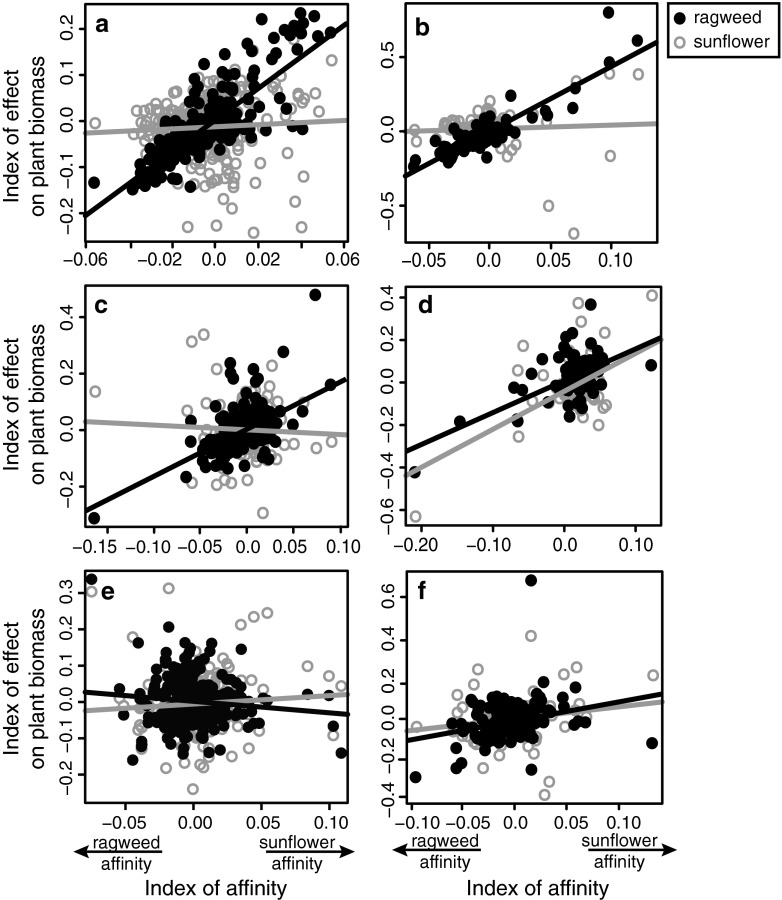
Affinity-effect relationships for microbial OTUs from representative trials. The selected trials are: **a** KS-2, bacteria; **b** KS-2, fungi; **c** MI-3, bacteria; **d** IL-2, fungi; **e** KS-1, bacteria; **f** KS-1, fungi. In each case, the points and trend lines representing the positive or negative effects of OTUs on the biomass of ragweed (*solid black*) and sunflower (*open gray*) are depicted in the same panel. We have arbitrarily oriented the axis of affinity such that microbial affinity for ragweed increases to the left, while affinity for sunflower increases to the right. Refer to Table 3 for slopes, significance levels, and *R* ^2^

We found a significant linear relationship between affinity and effect in 75 % of the trials, but the direction and strength of this relationship varied among the different trials and the different plants (Fig. 2, Table 3; Figs. S1, S2, S3, S4, S5, S6, S7 and S8). In some cases, the linear relationship provided a good fit to the data, e.g., ragweed in trial KS-2 (Fig. 2a,b, Table 3; Figs. S1, S4, S7 and S8), while some relationships generated a much lower *R* ^2^, indicating poor fit of the linear model for a majority of the microbial OTUs (Table 3). In the latter cases, poor model fit was sometimes due to a few highly influential OTUs at the extremes of the two indices (Fig. 2c,d; Figs. S2 and S3), and sometimes it was the result of a weak overall relationship (Fig. 2e,f; Fig. S5).

**Table 3:** Performance of affinity–effect models for ragweed and sunflower

		Ragweed		Sunflower	
Trial	Microbial group	Slope^a^	*R* ^2^	Slope^a^	*R* ^2^
IL-1	Bacteria	1.31***	0.30	0.40**	0.03
	Fungi	1.35***	0.24	0.78**	0.08
IL-2	Bacteria	1.59***	0.49	−0.97***	0.17
	Fungi	1.36***	0.30	1.33***	0.27
KS-1	Bacteria	−0.31 ns	0.01	0.23 ns	0.01
	Fungi	1.00***	0.08	0.70**	0.04
KS-2	Bacteria	3.26***	0.67	0.47*	0.02
	Fungi	4.14***	0.79	0.25 ns	0.00
MI-1	Bacteria	−1.04***	0.14	−0.23 ns	0.01
	Fungi	−1.94***	0.42	0.35 ns	0.02
MI-2	Bacteria	1.10***	0.14	−1.00***	0.10
	Fungi	2.11***	0.46	−0.40 ns	0.02
MI-3	Bacteria	1.66***	0.29	−0.17 ns	0.00
	Fungi	1.56***	0.30	−0.78**	0.08
MI-4	Bacteria	−0.43*	0.01	−0.67**	0.03
	Fungi	0.26 ns	0.00	−0.49*	0.01
OR	Bacteria	2.04***	0.43	−0.70***	0.04
	Fungi	2.20***	0.31	−0.38 ns	0.01
SD	Bacteria	1.58***	0.17	0.80***	0.07
	Fungi	0.44 ns	0.01	0.26 ns	0.01

^a^Significance codes: ***alpha <0.001; **alpha <0.01; *alpha <0.05; ns: alpha >0.05

In spite of this variability, a number of general patterns emerged. The affinity–effect relationship was more likely to be deemed significant and more likely to produce a better fit for ragweed than for sunflower (Table 3, Fig. 2). With the exception of the MI trials, most of the ragweed affinity–effect relationships showed a positive slope (Table 3), meaning that microbes with a high affinity for ragweed had the highest potential to harm its performance (and microbes with a low ragweed affinity potentially improved its performance). This is consistent with our earlier observation of widespread negative PSF with ragweed [36]. In contrast, the affinity–effect relationships for sunflower tended to have poor fit to the data and to produce positive and negative slopes in roughly equal measure (Fig. 2, Table 3). There were also more potentially important PSF OTUs for ragweed than for sunflower (Table 3). Across all trials, ragweed had an average of 2.6 more important bacterial OTUs (*p* = 0.039; *t* = 2.414, *df* = 9) and 1.7 more important fungal OTUs (*p* = 0.049; *t* = 2.278, *df* = 9) than sunflower.

## Discussion

In this study, we screened over 2,000 bacterial and 1,900 fungal OTUs for their potential to participate in PSF loops by assigning them indices of affinity and effect based on their occurrence in ten independent "home-and-away" trials. Because soil microbial communities are hyper-diverse [31], and because they respond to many different sources of environmental and biological selection [47, 48], it is often challenging to ascribe large portions of microbial community variability to simple gradients [48], such as plant-specific associations. We found that the largest source of microbial community variation in our study related to differences between the "home-and-away" trials (Fig. 1; Table 1), which we interpret as representing broad differences in the source pool of soil microorganisms available to participate in PSF. In light of this large variation in microbial source pools, it is not surprising that plants in this study were not able to consistently select for universal "ragweed" or "sunflower" microbial communities across all ten trials (Table 1). In this respect, our dataset is typical of many previous microbial ecology studies, where large-scale variability in community composition tends to dwarf any plant-related variation found across sites [49–52].

In addition to this background of source community variability, we encountered smaller-scale "noise" in the form of pot-to-pot variation in soil microbial community structure. Against this noise, we found that plant species identity was generally related to less than 20 % of the overall microbial community variability, as expressed in a single canonical axis (Table 2). This indicates that only a minority of microbial taxa expressed a detectable "affinity" for ragweed or sunflower. However, the indices of effect from this same minority of microbial taxa were able to account for over 70 % of the variability in plant biomass by partial least squares regression (Table 2). Thus, understanding the dynamics of this minor fraction of the microbial community may be sufficient to account for nearly all of the PSF potential in a plant–microbe system.

We were able to detect significant affinity–effect relationships in 75 % of the "home-and-away" trials (Table 3) in spite of the "signal-to-noise" challenges presented by large- and small-scale variation in microbial communities (Tables 1 and 2). We believe that this result provides good support for our hypothesis that an affinity-effect relationship exists for microorganisms with the potential to participate in PSF. Relatively low *R* ^2^ values in our models can be expected given that many of the microbes found in our "whole community" approach likely do not participate in PSF at all. In some cases, such as ragweed bacteria in MI-1, models had low R^2^ but several potentially important PSF bacteria were identified (Table 4). These taxa could be the targets of future cultivation efforts in order to characterize their ecology and perform controlled tests of their plant fitness effects.

**Table 4:** Potentially important^a^ OTUs in ragweed and sunflower plant–soil feedback

	Bacterial OTUs		Fungal OTUs	
Trial	Ragweed	Sunflower	Ragweed	Sunflower
IL-1	6	5	5	2
IL-2	3	1	1	3
KS-1	5	4	5	2
KS-2	13	2	7	4
MI-1	9	4	6	2
MI-2	6	6	5	3
MI-3	4	4	3	4
MI-4	3	3	2	1
OR	6	2	7	2
SD	5	3	2	3

^a^An OTU was deemed important if it was in the upper or lower 2.5 % tails of both affinity and effect indices for a given trial

The affinity–effect models performed better for ragweed than for sunflower, both in terms of the number of significant linear relationships (Table 3) and in the overall fit of the data (Fig. 2a,b, Table 3). We also found higher numbers of potentially important PSF OTUs for ragweed than for sunflower (Table 4). Furthermore, nearly every affinity–effect relationship for ragweed consistently indicated the potential for negative PSF. Bacteria and fungi with the highest affinity for ragweed were scored as being detrimental to ragweed biomass, and microbes with a high affinity for sunflower showed the potential to improve ragweed performance (Fig. 2, Table 3). Previous work with congeneric *Ambrosia artemisiifolia* (common ragweed) found that serial inoculations of ragweed-exposed soils results in a gradual decline in ragweed growth [53], suggesting the accumulation of soil pathogens in ragweed-dominated soils. Because giant ragweed is a common agricultural weed throughout the North Central region [54], it is possible that ragweed-specific soil pathogens have also been accumulating in the agricultural soils used in our trials, which may explain the consistent relationship between high-affinity, negative-effect ragweed microbes observed in our study (Table 3). As an alternative, the consistency of negative microbial feedback potential to ragweed across so many different source communities (Fig. 1) may indicate that ragweed is simply poorly defended against many forms of microbial attack and thus has a tendency to suffer from microbial interactions, in general. Negative PSF may be more common than positive feedback for a majority of plant species [55, 56], and it is considered to be a major driver of plant species coexistence and diversity [9, 19, 20]. If this is the case, then many plant species may be expected to generate the sorts of affinity–effect relationships in microbial community structure observed here for ragweed.

In contrast to those of ragweed, sunflower affinity–effect relationships were just as likely to indicate positive microbial feedback potential as negative, indicated by the changing signs of the slopes in Table 3. This may mean that sunflower PSF is highly dependent on the composition of the microbial source community (Fig. 1). However, this interpretation should be viewed with caution. Most of the regression coefficients for the sunflower affinity–effect relationships were very low, and so slopes that fluctuate around zero might also conservatively be interpreted as indicating no overall affinity–effect relationship for sunflower across the trials as a whole. Because we are using each of these trials as separate independent replicates, any differences between trials should be regarded as sampling error across the larger inference space of the North Central region. We do not want to try to diagnose trial-to-trial variability in this study any more than we would try to explain away pot-to-pot variation within a single trial. Nevertheless, the strength and direction of PSF may vary from location to location [57]. For example, non-native plants may experience positive or neutral PSF in their introduced ranges, while experiencing negative feedback in their native ranges [6, 10], and this pattern is sometimes explained by invoking differences in soil community composition between the native and introduced ranges, as per the enemy release hypothesis [58], and sometimes it is attributed to a lack of "familiarity" between plants and microbes in the new location [14]. Understanding how plant–microbe relationships vary across space to generate differential patterns of PSF is a promising area for future research, and our microbe-focused affinity–effect analysis could be fruitfully employed to identify the key microbial players in different locations.

While sunflower microbes did not show consistent affinity–effect relationships in regards to sunflower feedback potential, sunflower-associated microbes were generally classified by our analysis as potentially beneficial to ragweed (Fig. 2). The positive effect index of sunflower-affinity microbes was often just as high (or higher) for ragweed as it was for sunflower (Fig. 2a and b). This may indicate that sunflower is good at attracting generally beneficial soil microbes. Sunflower is thought to be a strong host for arbuscular mycorrhizal fungi [13], and it may be that sunflower improved the microbial condition for ragweed by enhancing mycorrhizal growth. We also found that ragweed often responded positively to sunflower-affinity bacteria (Fig. 2a), which would mean that sunflower may also condition the soil for plant growth-promoting rhizobacteria. We do not know the precise mechanism for sunflower-affinity microorganisms to positively affect ragweed, and controlled laboratory trials with specific isolates would be necessary to provide conclusive data, but our data supports Bever's idea that multiple plant species can interact through microbial PSF [11], leading to the maintenance or loss of plant species diversity. It is easy to envision that ragweed's consistent generation of negative PSF (Table 3) would tend to exclude ragweed from the system over time, but positive feedback from sunflower-affinity microbes might help overcome this tendency, contributing to coexistence of these two plants. The ultimate outcome of this multi-species interaction would depend on the relative effects of sunflower- and ragweed-affinity microbes on each plant.

## Conclusions

Feedback loops are of particular interest in community ecology because they allow a system to self-regulate. Negative feedback loops can lead to "homeostatic" stabilization, as exemplified by Bever's [11] PSF-mediated coexistence of plant competitors, and positive feedback loops can amplify small asymmetries resulting in dominance or exclusion of species. The results from our work showed that microorganisms are most likely to play a role in PSF loops when they possess an affinity for a particular plant and the capacity to strongly affect the growth of that plant. By examining the affinity–effect relationship against a background of diverse starting source communities, we were able to show broad support for this proposed relationship operating in microorganisms associated with ragweed and sunflower. The ragweed PSF system appears to enlist a broad subset of soil bacteria and fungi to produce consistently negative feedback, while the sunflower PSF may be contingent on interactions with a few key microorganisms. Our method provides a way to pinpoint key microbial players in PSF when confronted with the signal-to-noise challenge when searching for an unknown set of key microorganisms in communities that possess high alpha and beta diversity. While the ARISA method employed here does not provide specific information on the taxonomic identity of microbial OTUs, the use of our affinity–effect relationship with high-throughput DNA sequencing of microbial communities should help identify these potential key PSF microorganisms in future studies.

## Electronic supplementary material

Below is the link to the electronic supplementary material.

Fig. S1 (DOCX 451 kb)

Fig. S2 (DOCX 94 kb)

Fig. S3 (DOCX 292 kb)

Fig. S4 (DOCX 363 kb)

Fig. S5 (DOCX 138 kb)

Fig. S6 (DOCX 344 kb)

Fig. S7 (DOCX 284 kb)

Fig. S8 (DOCX 305 kb)
