# Supplementary material for: An Affinity–Effect Relationship for Microbial Communities in Plant–Soil Feedback Loops
Source: Microb Ecol. 2014 Jan 9;67(4):866–76. doi: 10.1007/s00248-013-0349-2 (PMC3984409; doi:10.1007/s00248-013-0349-2)
Supplement: Supplementary file 5 — (DOCX 138 kb) [file 248_2013_349_MOESM5_ESM.docx]

**Fig. S5**

**Figure S5.** Affinity-effect relationships for fungal OTUs from the MI-3 trial. Points and trend lines representing the positive or negative effects of OTUs on the biomass of ragweed (solid black) and sunflower (open gray) are depicted in the same panel. We have arbitrarily oriented the axis of affinity such that microbial affinity for ragweed increases to the left, while affinity for sunflower increases to the right. Refer to Table 3 for slopes, significance levels, and R^2^.
